# Individualisation of radiation protection recommendations for patients treated with [^177^Lu]Lu-DOTA-TATE

**DOI:** 10.1186/s40658-023-00570-7

**Published:** 2023-09-04

**Authors:** Teresa Monserrat Fuertes, Borja Santos Zorrozua, Emilia Rodeño Ortiz de Zarate, Miguel Ángel Peinado Montes, Carmen Vigil Díaz, Pablo Mínguez Gabiña

**Affiliations:** 1 Department of Medical Physics and Radiation Protection, Central University Hospital of Asturias, Oviedo, Spain; 2 Department of Surgery, Radiology and Physical Medicine, Faculty of Medicine and Nursing, UPV/EHU, Bilbao, Spain; 3 Scientific Coordination Unit, Biocruces Bizkaia Health Research Institute, Gurutzeta-Cruces University Hospital, Barakaldo, Spain; 4 Department of Nuclear Medicine, Biocruces Bizkaia Health Research Institute, Gurutzeta-Cruces University Hospital, Barakaldo, Spain; 5 Department of Nuclear Medicine, Central University Hospital of Asturias, Oviedo, Spain; 6 Department of Medical Physics and Radiation Protection, Biocruces Bizkaia Health Research Institute, Gurutzeta-Cruces University Hospital, Gurutzeta Plaza Z/G, 48903 Barakaldo, Bizkaia Spain; 7 Department of Applied Physics, Faculty of Engineering, UPV/EHU, Bilbao, Spain

**Keywords:** [^177^Lu]Lu-DOTA-TATE treatments, Radiation protection, Individualized recommendations

## Abstract

**Background:**

As for any other nuclear medicine treatment, patients treated with [^177^Lu]Lu-DOTA-TATE should be given some radiation protection recommendations after being discharged to limit the dose received by family members and public. The restriction periods will depend on the remaining activity at the time of discharge, the washout rate and patients’ personal conditions. The activity in patients’ whole-body follows a bi-exponential behaviour. At the time of discharge only the first part of the time-activity curve is known. However, the second phase of the bi-exponential curve should be known to individualize the time of restrictions. The main purpose of this prospective study was to establish a simple method for calculating the restriction periods based on measurements taken before discharge.

**Methods:**

The whole-body time-activity curve was calculated for 20 patients from dose-rate measurements performed during the first week post-administration. An effective decay time $T_{{{\text{eff}}}}^{6,24}$ was calculated from a mono-exponential fit performed with the 6 h and 24 h measurements and compared with the effective decay time $T_{{{\text{eff}}}}^{24,48,168}$ obtained from the mono-exponential fit performed with the 24 h, 48 h and 168 h measurements. The differences between them were calculated and the 95th percentile of these differences was used as a correction factor for $T_{{{\text{eff}}}}^{6,24}$. A modified effective decay $T_{{{\text{eff}},{\text{ mod}}}}^{6,24}$ was obtained by adding the correction factor to $T_{{{\text{eff}}}}^{6,24}$ and the restriction periods for each patient was calculated. The whole body activity washout between the first and the fourth treatment cycles of 16 patients was also compared.

**Results:**

The comparison of the whole-body activity curves between the first and the fourth cycle of the treatment for 16 patients would indicate that the recommendations on radiation protection determined from the first cycle could reasonably be used for the remaining cycles in most patients. The values of $T_{{{\text{eff}}}}^{6,24}$ and $T_{{{\text{eff}}}}^{24,48,168}$ obtained for the 20 patients were significantly different. The 95th percentile of the differences between $T_{{{\text{eff}}}}^{6,24}$ and $T_{{{\text{eff}}}}^{24,48,168}$ was 46 h, which is thus the time to be added to $T_{{{\text{eff}}}}^{6,24}$ so as to determine the restriction periods.

**Conclusions:**

The proposed method makes it possible to calculate the restriction periods for patients treated with [^177^Lu]Lu-DOTA-TATE before they leave the hospital in a conservative and individualized way.

## Background

The [^177^Lu]Lu-DOTA-TATE is a radiolabelled analogue of somatostatin used in the treatment of neuroendocrine tumours that are positive for receptors of that hormone. Treatment consists of the administration of four cycles of 7.4 GBq of the radiopharmaceutical, usually every 6–10 weeks [1]. The ^177^Lu has two main photopeaks at 113 keV and 208 keV with a relatively low photon emission yield of 6.20 and 10.38 photons per 100 decays, respectively [2]. Due to these gamma emissions it is necessary that patients—after being discharged—follow some recommendations that restrict their contact with members of the public and carers so that they do not receive effective doses that exceed the legal limits or recommended constraints [3, 4]. Some studies addressing radiation protection in treatments with [^177^Lu]Lu-DOTA-TATE have remarked on this necessity [5–9], and two of those studies reported fixed periods with recommendations that patients are told to follow [5, 6]. One of those studies [5] adopted a conservative approach and the longest restriction time periods calculated for a group of patients were set as recommendation for all patients. However, individualisation of recommendations would benefit those patients who could be given shorter time periods to follow the radiation protection recommendations. For instance, in the mentioned study [5] the range of days to restrict contact with partner was reported to vary between 3 and 15 d, and those patients with a faster excretion would benefit from individualisation of the radiation protection recommendations.

In order to provide patients with individualized recommendations on radiation protection to be applied after their discharge, it is necessary to have some knowledge of the pharmacokinetics of the [^177^Lu]Lu-DOTA-TATE in the whole body, which has been shown to follow a bi-exponential behaviour with a first fast component and a second slow component. The reported effective half-lives of those components are 1.3 h (0.9–1.5 h) and 50 h (45–57 h) [10], respectively. The excretion of the radiopharmaceutical occurs mainly through the urine, and most of this excretion occurs during the first day after treatment administration (approximately 64%) [11]. For this reason, some centres opt for treating patients as in-patients for 24 h after treatment administration, although the treatment could also be performed on an outpatient basis [5, 6, 12], depending on national legislation. Whole-body pharmacokinetics for individual patients could be derived from dose-rate measurements at a given distance from them and its determination would be influenced by the acquisition time-points at which those dose-rate values are acquired. Measurements to determine individualized radiation protection recommendations should ideally be acquired at time points belonging to the slow component of the washout, once the patient has been discharged. However, in the clinical practice, radiation protection recommendations are given at the time of discharge, and are thus obtained from time-point acquisitions belonging to the fast component and the early phase of the slow component, which may diminish the accuracy of the recommendations. Treating patients as in-patients for 24 h would help have some more information of the slow component of the washout before discharge.

At the Central University Hospital of Asturias, patients are admitted for 24 h after treatment administration and currently all of them receive at discharge the same recommendations on radiation protection. These recommendations consist of avoiding close contact with children and pregnant women, sleeping alone in the room and staying off work for a period of 10 d. In this study, different aspects related to radiation protection recommendations were addressed. First, a comparison of the washout of the whole-body activity between the first and the fourth cycle was performed in order to study the possibility of using the radiation protection recommendations determined in the first cycle in the remaining three cycles. Second, the individualisation of those recommendations was addressed, including an optimisation of the data collection necessary to calculate them. To this end, the whole-body activity was measured up to one week after administration of [^177^Lu]Lu-DOTA-TATE. From the whole-body time-activity curve of each patient, effective half-lives of the [^177^Lu]Lu-DOTA-TATE in the whole-body were determined from dose-rate measurements acquired during in-patient time and after discharge. The radiation protection recommendations obtained using the latter dose-rate measurements would be more accurate, but patients are provided with instructions at their release, approximately at 24 h post-administration. This study aimed to find a procedure to determine accurate radiation protection recommendations based on the dose-rate measurements carried out up to 24 h post-administration.

## Method

### Patient data

This prospective study—performed at the Central University Hospital of Asturias—included the twenty patients (14 male and six female), aged between 31 and 79 years, treated between 2019 and 2022 for neuroendocrine tumours with [^177^Lu]Lu-DOTA-TATE. The treatment consisted of four cycles, in each of which 7.4 GBq of radiopharmaceutical were administered. After treatment administration, patients remained as in-patients for 24 h. Patients were released when the dose rate at 1 m was below 20 μSv/h, this value being adapted from the recommendations of the document of the European Commission for Radiation Protection following iodine-131 therapy [13]. Institutional Ethics Committee approval was obtained (reference code: CEImPA 2022.313), as well as informed consent from all patients.

### Radiation protection recommendations

When a patient is discharged after being treated with [^177^Lu]Lu-DOTA-TATE, the effective dose received by people in contact with the patient must not exceed the legal limits or the recommended constraints [3, 13]. This can be achieved by establishing a restriction period, ${T}_{r}$, to follow recommendations based on radiation protection which are given to patients when they are discharged. In this study, the following radiation protection recommendations-based on those appearing in the IAEA report 63 [14]—are set to give to patients after they are discharged:

- Avoid spending periods > 3 h at < 1 m and sleep away from partner until…
- Avoid spending periods > 3 h at < 1 m with other adults at home until…
- Avoid spending periods > 3 h at < 1 m with children at home until…
- Avoid spending periods > 3 h at < 1 m with other persons away from home until…
- You may return to work on…
- Recommended times in public transport in following days after discharge…

Before being given these radiation protection recommendations, patients must respond to a questionnaire in which they specify cohabitants and work situation. Some of those recommendations would be omitted if they are not applicable (e.g. if the patient is not working at the time of discharge).

The effective dose $D$ received by people in contact with the patient can be estimated by integrating over time the measured dose rate and taking into account the time that those people will spend at different distances from the patient throughout the day. $D$ is the sum of two definite integrals: one from the time of discharge to the end of the restriction period and another one from the end of the restriction period to infinity. If the patient were considered to be a point source, the effective dose $D$ would be given by:

1 $$D = \frac{{\dot{D}_{1m} }}{24}\frac{{\left( {1 - 2^{{ - T_{r} /T_{{{\text{eff}}}} }} } \right)}}{{{\text{ln}}2/T_{{{\text{eff}}}} }}\frac{{t_{{{\text{d}}r}} }}{{d_{r}^{2} }} + \frac{{\dot{D}_{1m} }}{24}\frac{{2^{{ - T_{r} /T_{{{\text{eff}}}} }} }}{{{\text{ln}}2/T_{{{\text{eff}}}} }}\mathop \sum \limits_{i} \frac{{t_{{{\text{dnr}},i}} }}{{d_{{{\text{nr}},i}}^{2} }}$$

where ${\dot{D}}_{1m}$ is the dose rate at 1 m from the patient at discharge time, ${T}_{\mathrm{eff}}$ is the effective half-life of the slow component of the [^177^Lu]Lu-DOTA-TATE in the whole-body, ${t}_{\mathrm{dr}}$ is the number of hours per day that people in contact with patient are assumed to be at a distance ${d}_{r}$ during the time period to follow the recommendations, and ${t}_{\mathrm{dnr}}$ is the estimated number of hours per day that people in contact with patient will spend at a distance ${d}_{\mathrm{nr}}$ after the time period with recommendations, and the summation refers to different time periods. However, the use of ${\dot{D}}_{1m}$ in Eq. 1 to determine the dose rates at distances shorter than 1 m by applying the inverse square law would be inaccurate, as the patient cannot be considered a point source. Therefore, factors that relate the dose rate at 1 m to shorter distances—${f}_{\mathrm{dr}}$ during the restriction time period or ${f}_{\mathrm{dnr},i}$ after that time period—and that can be determined from patients’ measurements were introduced in Eq. 1 which becomes:

2 $$D = \frac{{\dot{D}_{1m} }}{24}\frac{{\left( {1 - 2^{{ - T_{r} /T_{{{\text{eff}}}} }} } \right)}}{{{\text{ln}}2/T_{{{\text{eff}}}} }}f_{{{\text{d}}r}} t_{{{\text{d}}r}} + \frac{{\dot{D}_{1m} }}{24}\frac{{2^{{ - T_{r} /T_{{{\text{eff}}}} }} }}{{{\text{ln}}2/T_{{{\text{eff}}}} }}\mathop \sum \limits_{i} f_{{{\text{dnr}},i}} t_{{{\text{dnr}},i}}$$

The time to follow radiation protection recommendations can thus be obtained by solving ${T}_{r}$ in Eq. 2 and establishing a limit or a constraint for the effective dose, ${D}_{l/c}$, for the corresponding population group:

3 $$T_{r} = - T_{{{\text{eff}}}} \log_{2} \left[ {\frac{{\frac{{24\ln 2D_{l/c} }}{{T_{{{\text{eff}}}} \dot{D}_{1m} }} - f_{{{\text{d}}r}} t_{{{\text{d}}r}} }}{{\mathop \sum \nolimits_{i} f_{{{\text{dnr}},i}} t_{{{\text{dnr}},i}} - f_{{{\text{d}}r}} t_{{{\text{d}}r}} }}} \right]$$

Therefore, the data obtained from patients’ measurements needed to calculate the radiation protection recommendations are: ${\dot{D}}_{1m}$, the dose rate at 1 m from the patient at discharge time, and ${T}_{\mathrm{eff}}$, the effective half-life of the slow component of the [^177^Lu]Lu-DOTA-TATE in the whole-body.

An Excel spreadsheet (Microsoft Corporation 2007) [15] was developed to calculate the restriction periods for each recommendation which will appear in an individualized card to be given to each of the patients after they are discharged. Note that due to the presence of the logarithm in Eq. 3, there will be situations where the equation has no solution or returns a solution that does not make physical sense. On the one hand, for low enough values of ${T}_{\mathrm{eff}}$ and/or ${\dot{D}}_{1m}$, that is, for patients with a fast clearance, the value of the logarithm will be positive and, consequently, ${T}_{r}$ will be a negative number. On the other hand, for high enough values of ${T}_{\mathrm{eff}}$ and/or${\dot{D}}_{1m}$, that is, for patients with slow clearance, the logarithm of a negative number will be obtained, in which case Eq. 3 would not have a solution. For the former scenario, the value of ${T}_{r}$ would be set to 0, and for the latter scenario, the number of hours per day in the restriction period would be set to 0, that is, in the restriction period the patient should be as much isolated as possible. To be conservative, all ${T}_{r}$ results were rounded to the next higher whole number.

### Measurement of the whole body pharmacokinetics

Six whole-body dose-rate measurements were performed at approximately 0 h, 2 h, 6 h, 24 h, 48 h and 168 h after at least one treatment cycle for the 20 patients. Data from the first and the fourth treatment cycles were included for 16 of the 20 patients. For two patients only data from the first cycle were available, as those patients could not finish the treatment, and for other two patients only data from the fourth cycle were available, as their data from the first cycle could not be acquired. Measurements were performed with a pressurized ion chamber survey meter Victoreen 450P (Solon, Ohio, USA) at a distance of 1 m from the patient and at the height of the xiphoid appendix. To account for the redistribution of activity within patient's body, two measurements were taken at each time point—one in the anteroposterior position and another one in the posteroanterior position—and the geometric mean was calculated [16]. The signal-to-background ratio was above 40 in all measurements and therefore, none of the measurements was corrected for background. The first measurement was performed immediately after the administration of the radiopharmaceutical and before the patient had excreted any activity. This way, the measured dose rate at each time could be normalized to the activity administered to the patient, which was calculated by measuring the filled and empty [^177^Lu]Lu-DOTA-TATE vial on a Capintec CRC-55tR activity meter (Florham Park, New Jersey, USA) and correcting for the decay of ^177^Lu. The remaining activity in the catheters and needles used for administration was ignored. Additionally, dose-rate measurements were performed at 0.1 m and 0.5 m in order to determine the factors introduced in Eq. 2 that relate the dose rate at 1 m to those distances.

### Effective dose limits and recommended constraints

For adults cohabiting with patients, excluding pregnant women, the effective dose constraints for carers and comforters recommended by the European Commission [13] were applied. For all other cases, the effective dose limit for members of the public given by the Spanish law [17] was used. Treatment with [^177^Lu]Lu-DOTA-TATE consists of four cycles administered every 6–10 weeks, so those values were divided by a factor 4, except for the case of travelling at public transport. Table 1 specifies the limits and constraints considered for each treatment cycle for the different population groups.

**Table 1:** Effective dose limits and constraints considered for each population group

Group of population	Limit/constraint (mSv/cycle)
Children; pregnant women; people away from home	0.25
Adults < 60 years old at home	0.75
Adults > 60 years old at home	3.75
Public transport users	1

### Assumptions on patients’ contact with other people

During the time period with restrictions, it is assumed that patients may spend 3 h per day at 1 m from other people at home, as well as away from home. Patients must not return to work until this period ends. After being discharged, they can use public transport for a time dependent on the dose rate at 0.1 m. To obtain recommendations to be given to patients, the following assumptions were made regarding patients’ contact with others after the period of restricted-contact recommendations ends [18–20].

- at home:
  patients will spend 6 h at 1 m and 3 h at 0.5 m from any adult and another 8 h at 0.1 m with partner when sleeping together.
  patients will spend 15 periods of 35 min per day at 0.1 m from infants of less than 2 years.
  patients will spend 8 h at 1 m and 4 h at 0.1 m from children between 2 and 5 years.
  patients will spend 4 h at 1 m and 2 h at 0.1 m from children between 5 and 14 years.
- away from home:
  patients will spend 3 h at 1 m, 2 h at 0.5 m and 1 h at 0.1 m from any person, adult or child.
- at work:
  patients will spend 8 h at 1 m from adult co-workers.
  patients will spend 4 h at 1 m, 2 h at 0.5 m and 2 h at 0.1 m from children (if working with them).
- on public transport (this assumption applies after patient is discharged):
  patients will travel at a distance of 0.1 m from other passengers.

### Graphical representation and statistical analysis

Graphical representation was performed with Matplotlib [21] and R (R 4.1.3; R Foundation for Statistical Computing, Vienna, Austria) [22], which was also used together with SciPy [23] to perform the statistical analysis.

The distributions of the effective half-lives were tested for normality using the Shapiro-Wilks test, for which *p* < 0.05 was considered non-normal. If the distribution were non-normal, a two-tailed Mann–Whitney *U* test would be performed to compare results obtained from different methods and, if normal, an independent *t*-test. A value of *p* < 0.05 was considered significant. Moreover, the uncertainty of the effective half-lives was calculated and also the uncertainty in the evaluation of the 95th percentile [24].

## Results

### Whole-body pharmacokinetics in the first and the fourth cycles of the treatment

**Fig. 1:**
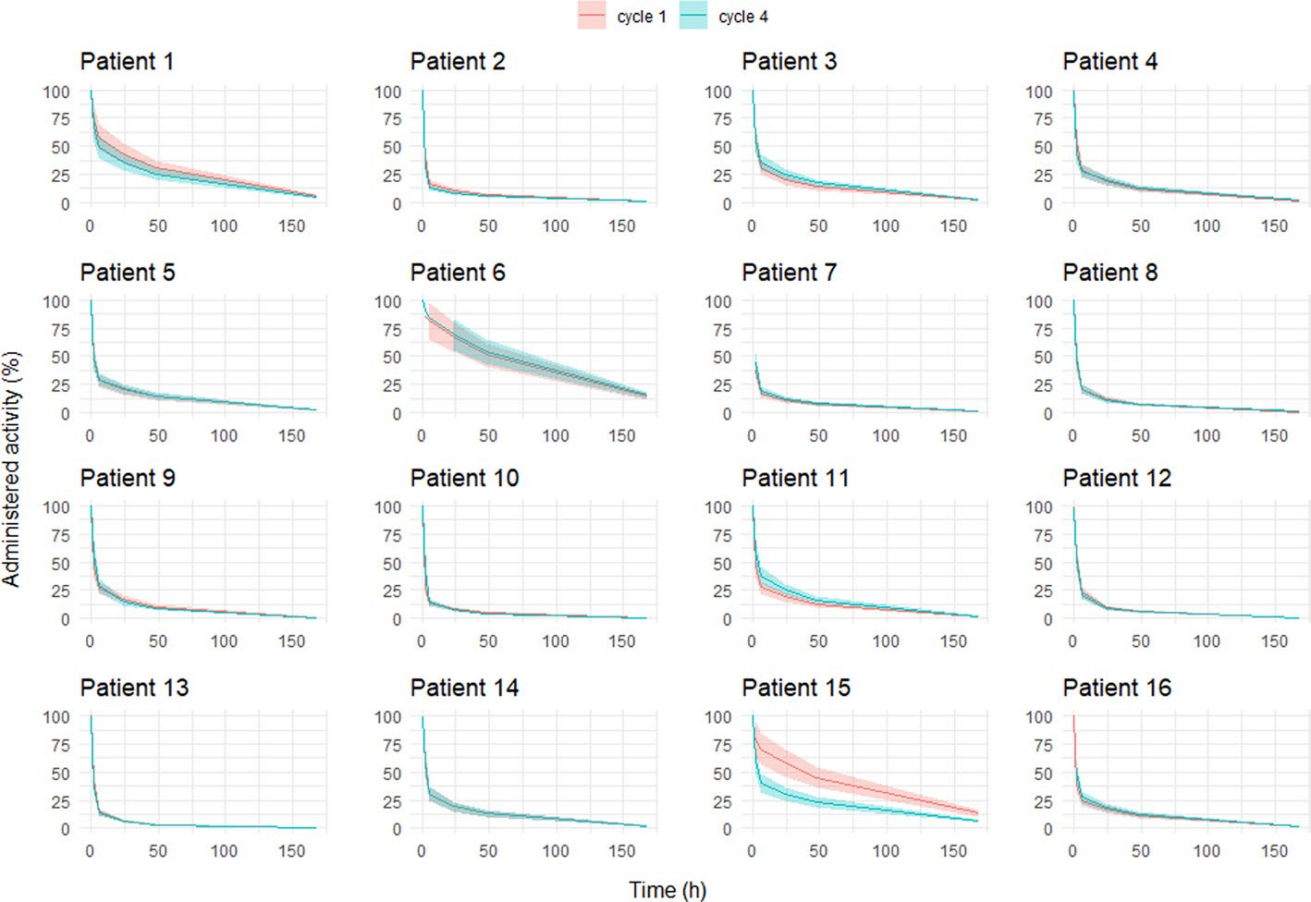
Curves of the remaining whole-body activity as a function of time for the first and the fourth cycle corresponding to the 16 patients for whom those data were available, including an uncertainty in the remaining whole-body activity of 20%

First, in order to address the possibility of determining the restriction periods from the pharmacokinetics measured during the first cycle for the rest of the cycles, a comparison between the first and the fourth cycle of the curves of the remaining whole-body activity in percentage, ${A}_{\mathrm{wb}}(\%)$, as a function of the time post-administration was performed for 16 patients. Bi-exponential curve fitting by the least-squares method was performed using the six dose-rate measurements for each patient and treatment cycle. The uncertainty in the measured values of ${A}_{\mathrm{wb}}$ was estimated by error propagation to be of 20%, for uncertainties of 10% in the activity of the vial measured in the activity meter; of 10% in the reading of the measurements with the ion chamber survey meter; and of 5% in the distance between the ion chamber survey meter and the patient. For each of the sixteen patients, curves of ${A}_{\mathrm{wb}}$ were represented as a function of time for the first and the fourth cycle including the uncertainty of 20% (Fig. 1). It can be noted that for all patients except one (patient 15) both curves overlap, which indicates that the recommendations on radiation protection determined from the first cycle could reasonably be used for the remaining cycles.

### Determination of the radiation protection recommendations from dose-rate measurements before patients’ discharge

**Fig. 2:**
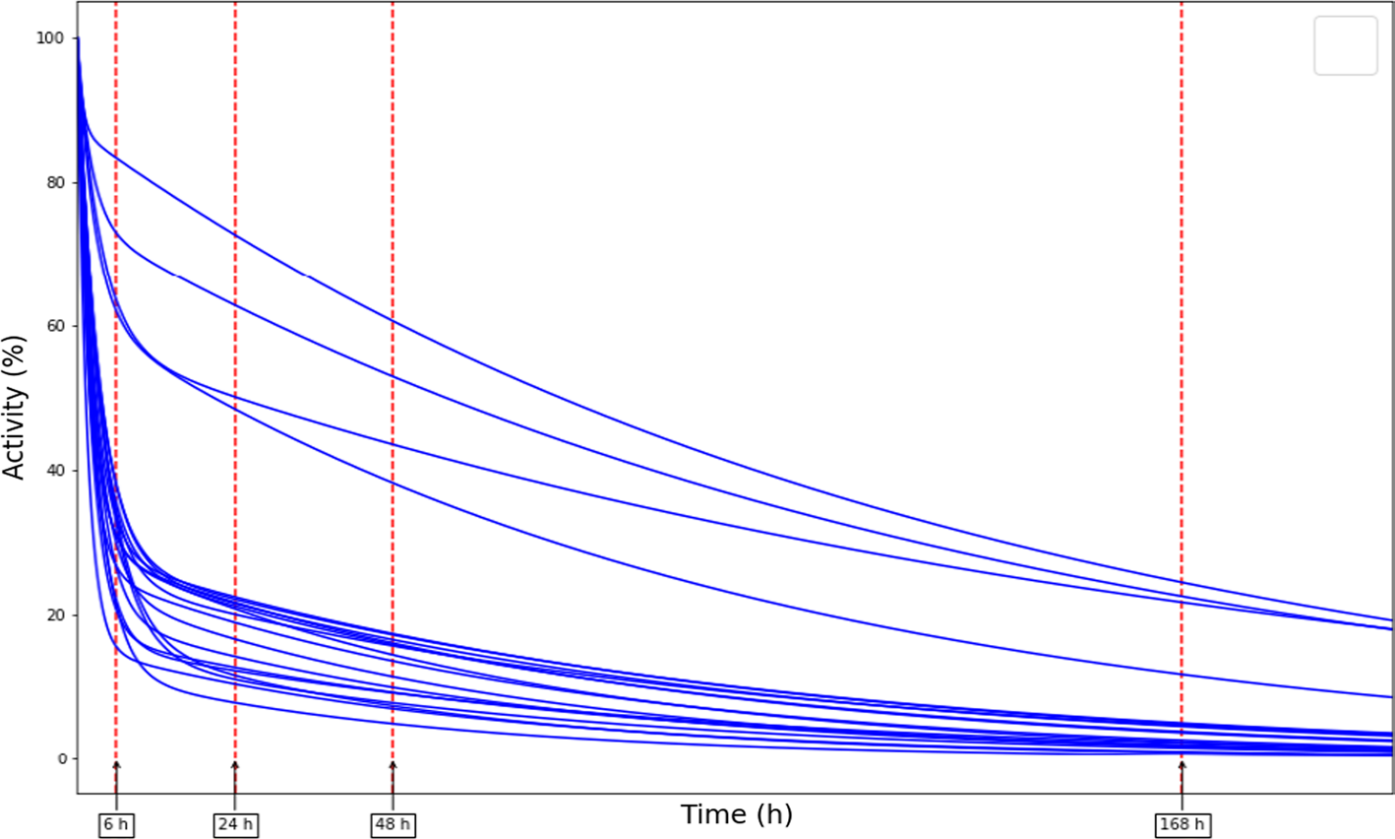
Percentage of the remaining whole-body activity $A_{{{\text{wb}}}} \left( \% \right)$ as a function of time for the 20 patients included in the study

Curves of the remaining whole-body activity in percentage, ${A}_{\mathrm{wb}}(\%)$, as a function of time post-administration were analysed for 20 patients in total. For 18 patients data belonged to the first cycle of the treatment, and for two patients to the fourth cycle, as it was not possible to acquire measurements in the first cycle. Including data of those two patients seems reasonable based on the results of the previous section. Figure 2 shows the obtained curves, which were derived from the six dose-rate measurements at approximately 0 h, 2 h, 6 h, 24 h, 48 h and 168 h acquired at 1 m from the patient. To facilitate subsequent calculations and comparisons, the exact points of 6 h, 24 h, 48 h and 168 h were calculated on each curve. The bi-exponential nature of the activity washout, with a fast and a slow component, can be noted, and also the notable variability of the washout among some of the patients.

Patients were treated as inpatients during the first 24 h after treatment administration until they were discharged. The median dose rate at the moment of discharge was 5.4 μSv/h. Minimum, 1st quartile (Q1), 3rd quartile (Q3) and maximum values were 2.4 μSv/h, 3.7 μSv/h, 6.5 μSv/h and 19.5 μSv/h, respectively. Differences between the effective half-lives of the slow components of the bi-exponential curves in Fig. 2 and the effective half-lives determined from the whole-body activity values at 24 h, 48 h and 168 h, $T_{{{\text{eff}}}}^{24,48,168}$, were < 0.4%. Those values of $T_{{{\text{eff}}}}^{24,48,168}$ would describe the whole-body pharmacokinetics after patients’ discharge and should thus lead to accurate radiation protection recommendations using Eq. 3. However, those recommendations should preferably be given to patients at the moment in which they are discharged and, therefore, a value for the ${T}_{\mathrm{eff}}$ using whole-body activity values determined during the first 24 h post-administration should be used.

Table 2 shows the values of the effective half-lives considering a mono-exponential curve for the whole-body activity washout derived from values at 6 h and 24 h, ${T}_{\mathrm{eff}}^{\mathrm{6,24}}$, and from values at 24 h, 48 h and 168 h, $T_{{{\text{eff}}}}^{24,48,168}$, for the 20 patients considered in this section. The Shapiro-Wilks test showed that neither $T_{{{\text{eff}}}}^{24,48,168}$ nor ${T}_{\mathrm{eff}}^{\mathrm{6,24}}$ followed a normal distribution. The median, the minimum, Q1, Q3 and the maximum values are also shown in Table 2. The Mann–Whitney test showed that the median values of both parameters, ${T}_{\mathrm{eff}}^{\mathrm{6,24}}$ and $T_{{{\text{eff}}}}^{24,48,168}$, were significantly different (*p* < 0.05).

**Table 2:** Effective half-lives obtained from 6 and 24 h, $T_{{{\text{eff}}}}^{6,24}$, and from 24 h, 48 h and 168 h, $T_{{{\text{eff}}}}^{24,48,168}$, for the patients included in the study. The median (minimum, Q1, Q3, maximum) values are also shown

Patient	$T_{{{\text{eff}}}}^{6,24}$ (h)	$T_{{{\text{eff}}}}^{24,48,168}$ (h)
1	23 ± 6	68 ± 6
2	51 ± 29	70 ± 7
3	21 ± 5	58 ± 4
4	23 ± 6	67 ± 6
5	24 ± 7	58 ± 4
6	32 ± 11	65 ± 6
7	92 ± 95	92 ± 11
8	26 ± 8	53 ± 4
9	20 ± 5	47 ± 3
10	53 ± 32	119 ± 19
11	21 ± 5	44 ± 3
12	32 ± 11	45 ± 3
13	31 ± 11	42 ± 2
14	35 ± 14	54 ± 4
15	12 ± 2	50 ± 3
16	12 ± 2	38 ± 2
17	13 ± 2	35 ± 2
18	22 ± 6	64 ± 5
19	84 ± 79	97 ± 12
20	40 ± 18	57 ± 4
	25 (12, 21, 36, 92)	58 (35, 47, 67, 119)

**Fig. 3:**
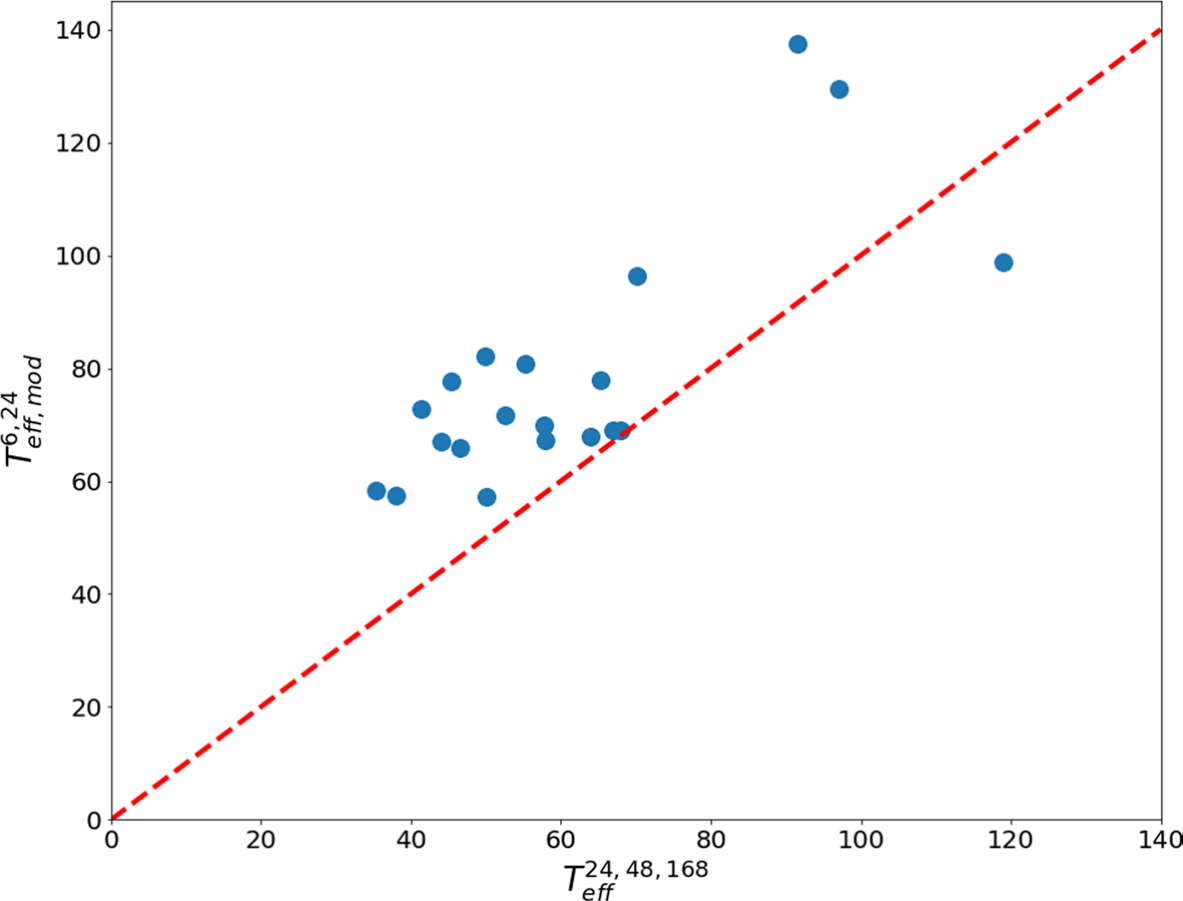
Values of $T_{{{\text{eff}}, {\text{mod}}}}^{6,24}$ as a function of values of $T_{{{\text{eff}}}}^{24,48,168}$. The dashed line represents the line of identity

Taking into account that ${T}_{\mathrm{eff}}^{\mathrm{6,24}}$ values are significantly lower than ${T}_{\mathrm{eff}}^{\mathrm{24,48,168}}$ values, using ${T}_{\mathrm{eff}}^{\mathrm{6,24}}$ to determine the restriction periods in Eq. 3 would underestimate those periods, as the pharmacokinetics for the whole-body activity after patients are discharged is better described by the values at 24 h, 48 h and 168 h. In order to derive a method to give patients radiation protection recommendations obtained using ${T}_{\mathrm{eff}}^{\mathrm{6,24}}$, the 95th percentile of the differences between ${T}_{\mathrm{eff}}^{\mathrm{6,24}}$ and $T_{{{\text{eff}}}}^{24,48,168}$ was calculated as well as the uncertainty, and values of 46 h and 4 h were, respectively, obtained. Then, the 95^th^ percentile was added to ${T}_{\mathrm{eff}}^{\mathrm{6,24}}$, resulting in a modified effective half-life that will further be referred to as $T_{{{\text{eff}}, {\text{mod}}}}^{6,24}$. The restriction periods calculated by using $T_{{{\text{eff}}, {\text{mod}}}}^{6,24}$ as the effective half-life in Eq. 3 should be a reasonable and conservative surrogate of the restriction periods calculated with $T_{{{\text{eff}}}}^{24,48,168}$. Figure 3 shows the values of $T_{{{\text{eff}}, {\text{mod}}}}^{6,24}$ as a function of values of $T_{{{\text{eff}}}}^{24,48,168}$. It can be noted that $T_{{{\text{eff}}, {\text{mod}}}}^{6,24}$ values are higher than $T_{{{\text{eff}}}}^{24,48,168}$ values for all patients but one. This was to be expected, since it is a direct consequence of having chosen the 95^th^ percentile of the differences as the correction factor.

**Fig. 4:**
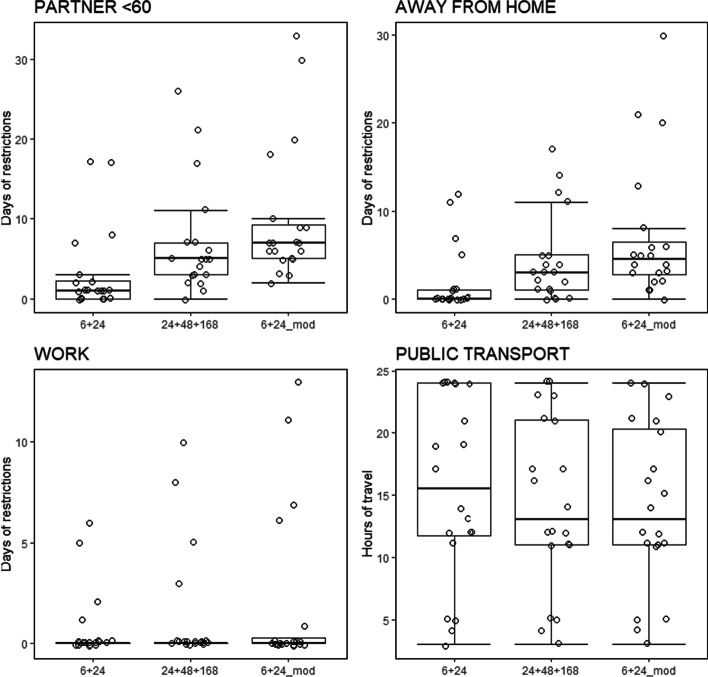
Box plots of the restriction periods using $T_{{{\text{eff}}}}^{6,24}$, $T_{{{\text{eff}}}}^{24,48,168}$ and $T_{{{\text{eff}}, {\text{mod}}}}^{6,24}$ obtained for patients included in the study and assuming that patients lived with a partner < 60 y who was considered as a carer, and worked at a shared office. Symbols show individual patient values and have been randomly displaced in the horizontal direction for improved visibility. Note that in the calculation of hours of travel all results greater than 24 h have been set to 24 h

Lastly, in order to compare the restriction periods determined by using ${T}_{\mathrm{eff}}^{\mathrm{6,24}}$, $T_{{{\text{eff}}}}^{24,48,168}$ and $T_{{{\text{eff}}, {\text{mod}}}}^{6,24}$, those time periods were calculated using the values for the 20 patients included in the study. Regarding personal conditions, it was assumed that patients lived with a partner < 60y who was considered as a carer, and worked at a shared office. To perform calculations, distance correction factors (${f}_{\mathrm{dnr},i}$) were calculated for distances of 0.1 m and 0.5 m from median values of the dose-rate measurements performed for all patients at 0.1 m, 0.5 m and 1 m. The results obtained were ${f}_{0.1m}=14.9$ and ${f}_{0.5m}=2.5$. Note that the distance correction factor ${f}_{\mathrm{dr}}$ equals 1 because the assumed contact distance for the time period with restrictions is 1 m. Figure 4 shows box plots of the time periods with restrictions for the abovementioned radiation protection recommendations. For the assumed personal conditions, recommendations 2 and 3 would not apply. For the recommendations on public transport the maximum recommended time in public transport in the first day after discharge is given. It can be observed that results obtained using $T_{{{\text{eff}}, {\text{mod}}}}^{6,24}$ tend to be more conservative than those obtained using $T_{{{\text{eff}}}}^{24,48,168}$, and that the proposed method allows for individualisation of the recommendations given.

### Individualized card with recommendations for the patient

**Fig. 5:**
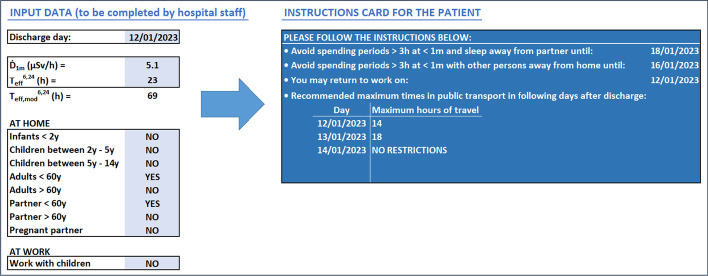
Example of spreadsheet with input data and card with recommendations for the patient

Figure 5 shows the spreadsheet created to calculate the restriction periods, as well as the individualized card with recommendations that would be given to patients after they are discharged. Once the patients have answered a questionnaire regarding their personal contacts, individualized recommendations are determined using the calculated value of $T_{{{\text{eff}}, {\text{mod}}}}^{6,24}$ and a personal card including those recommendations is generated. The example shown in Fig. 5 is based on data for patient 1 and the personal conditions used to determine the box plots in Fig. 4.

## Discussion

In this study, the delivery of individualized radiation protection recommendations to patients treated with [^177^Lu]Lu-DOTA-TATE after being discharged has been addressed. A method to obtain those recommendations using the effective half-life of the [^177^Lu]Lu-DOTA-TATE activity in the whole-body calculated from dose-rate measurements at 6 h and 24 h post-administration, ${T}_{\mathrm{eff}}^{\mathrm{6,24}}$, has been developed. Those time points are convenient from a practical viewpoint, especially if patients remain as in-patients the first 24 h after treatment administration. However, as the whole-body washout after patients leave the hospital gets slower, the use of that effective half-life would underestimate the restriction time periods. The effective half-life obtained from dose-rate measurements at 24 h, 48 h and 168 h, $T_{{{\text{eff}}}}^{24,48,168}$, would lead to more accurate radiation protection recommendations, but this procedure would not be feasible in the clinical routine, as recommendations should be given before patients’ contact with members of the public and carers starts.

The recommendations obtained following the procedure developed may not be as accurate as those determined from the 24 h, 48 h and 168 h time points but, according to the results shown, they would tend to be more conservative and still individualized instructions. Due to the methodology applied in this study which uses the 95^th^ percentile to calculate the correction factor, there would be a 5% of patients for whom the time of restrictions calculated with $T_{{{\text{eff}}, {\text{mod}}}}^{6,24}$ would be lower than that obtained with $T_{{{\text{eff}}}}^{24,48,168}$. In those cases there is a possibility that the dose limit or constraint may be exceeded. However, it must be taken into account that the contact times and distances were chosen conservatively. A permanent monitoring of the patients after patients are discharged to see if they follow the radiation protection recommendations is not feasible, but a recent study [25] showed that the effective dose received by the partner of the patient was below the recommended constraint, which would mean that the proposed methodology could still be quite effective for that 5% of patients.

Regarding the calculation of the 95th percentile of the differences between the ${T}_{\mathrm{eff}}^{\mathrm{6,24}}$ and the ${T}_{\mathrm{eff}}^{\mathrm{24,48,168}}$ values, it is worth noting that for a discontinuous data distribution there are three methods to determine a percentile calculation [26]. In two of the three methods the 95th percentile obtained is of 46 h and in the third method of 65 h. The third method has some limitations when the number of data is small, as is the case of this study. We thus used the 95th percentile obtained from the first two methods. As the number of data increases the three methods would produce similar results but the number of patients included in the study was limited by the low number of cases treated with [^177^Lu]Lu-DOTA-TATE at the Central University Hospital of Asturias. Further studies including a greater number of patients are thus warranted. The difference between both percentiles is lower than 1 d. The use of a 95th percentile of 65 h would have been a more conservative approach, but a recent study [25] showed that patients tended to be conservative in relation to the radiation protection recommendations and that would mean that in the practice they are following recommendations obtained with an effective half-life greater than 46 h.

In case that the treatment is administered on an outpatient basis, if the method developed in this study is going to be implemented, patients should go back to the hospital to acquire the 24 h dose-rate measurement. In that case, patients would be recommended to avoid any contact with others during those hours. The first dose-rate measurement would be performed when patients are discharged from the hospital, once the infusion of the amino acids, which lasts between 4 and 6 h [27], is finished. Patient imaging is normally performed for dosimetry or to see the distribution of the [^177^Lu]Lu-DOTA-TATE, and it could be scheduled at 24 h post-administration (when there are not dead-time effects) in order to perform the second dose-rate measurement.

The comparison of the curves of the remaining whole-body activity in percentage as a function of time corresponding to the first and the fourth cycles showed that for all patients but one both curves overlapped when the uncertainty in the activity values is considered. Therefore, it seems reasonable that the restriction periods obtained in the first cycle could be applied to the following cycles. However, the fact that one outlier was observed indicates that a measurement at 6 h is advisable in all cycles in order to check for differences in the pharmacokinetics and if so, a measurement at 24 h should be performed in order to calculated the restriction periods with the methodology described in this study.

The radiation protection recommendations given try to cover the situations in which the patients will be in regular contact with other people, which include people cohabiting with patients (partner, children or other adults), people not cohabiting but having regular contact with patients, working colleagues or children (if for instance patients work at a nursery) and those people that patients may be close to at public transport. Restriction periods for food manipulation have been included in some studies, recommending a restriction period of 3 days [6] or a day extra added to the restriction period before returning to work [18]. If patients leave the hospital at an ambulance, they could spend longer times than those recommended for public transport, as the distance between the patient and the driver would be bigger.

**Fig. 6:**
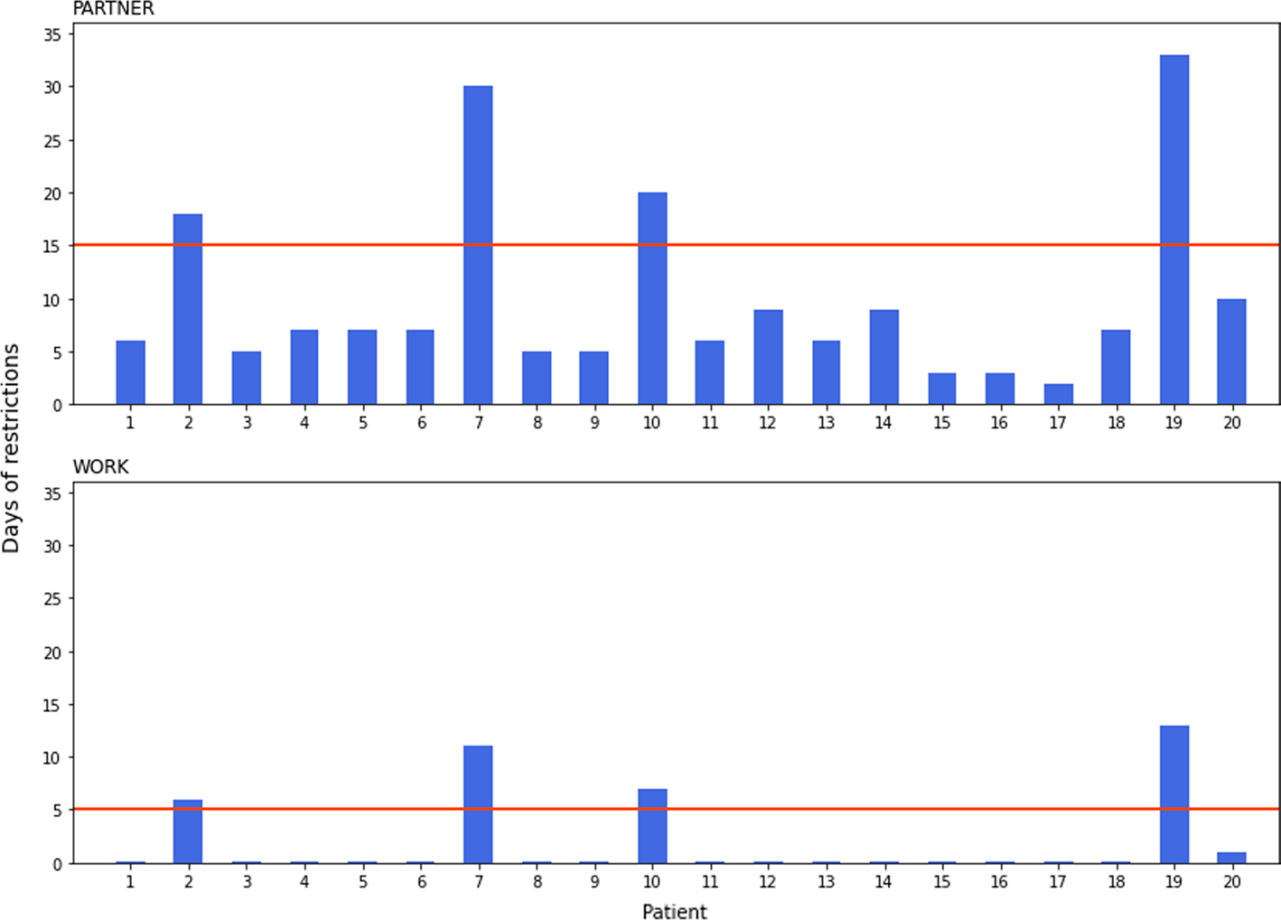
Comparison of the individualized recommendations obtained following the method developed in this study (blue bars) with those obtained in the study by Levart et al. [5] (horizontal red line) for the case of a partner < 60 y (top) and for the case of days off work (bottom)

Lastly, Fig. 6 shows a comparison of the individualized recommendations obtained following the method developed in this study with the recommendations obtained in the study by Levart et al. [5], those recommendations being fixed restriction periods obtained as the 95th percentile of the restriction periods obtained for a group of patients. The comparison was performed for the case of a partner < 60 y and for the case of days off work. In our study, the effective dose limits considered were more conservative than in the study by Levart et al. [5], (0.75 mSv/cycle for family members and 0.25 mSv/cycle for co-workers versus 1 mSv/cycle for family members and 0.3 mSv/cycle for co-workers) and the same occurred with the patients’ contact time with partner, as in our study an additional contact time of 3 h at 0.5 m was considered. The benefit of the individualisation of the recommendations is thus outstanding. On the one hand, for most patients the restriction periods are shorter than the generalized recommendations, which will make patients’ life easier, in addition to reduce the private and social costs of limiting contact with other people and staying off work. On the other hand, for the case of the patients in which those periods are longer, members of the public and carers would benefit, as they will assure that the effective doses are within the legal and recommended limits.

## Conclusion

Recommendations of radiation protection are necessary for patients treated with [^177^Lu]Lu-DOTA-TATE. A method to determine those recommendations for each individual patient has been developed using the dose-rate measurements at 6 h and 24 h. Individualisation of the recommendations implies that most patients can return to their normal life earlier than if they receive fixed restrictions. It also brings safety to carers and members of the public in those cases in which the periods of recommendations are longer than in the generalized recommendations.

## Data Availability

Data can be provided upon a reasonable request to the corresponding author.
